# Frequency sensitive mechanism in low-intensity ultrasound enhanced bioeffects

**DOI:** 10.1371/journal.pone.0181717

**Published:** 2017-08-01

**Authors:** April D. Miller, Abdoulkadri Chama, Tobias M. Louw, Anuradha Subramanian, Hendrik J. Viljoen

**Affiliations:** 1 Department of Chemical and Biomolecular Engineering, University of Nebraska-Lincoln, Lincoln, Nebraska, United States of America; 2 Department of Chemical Engineering, Stellenbosch University, Stellenbosch, South Africa; University of Hawai'i at Manoa, UNITED STATES

## Abstract

This study presents two novel theoretical models to elucidate frequency sensitive nuclear mechanisms in low-intensity ultrasound enhanced bioeffects. In contrast to the typical 1.5 MHz pulsed ultrasound regime, our group previously experimentally confirmed that ultrasound stimulation of anchored chondrocytes at resonant frequency maximized gene expression of load inducible genes which are regulatory markers for cellular response to external stimuli. However, ERK phosphorylation displayed no frequency dependency, suggesting that the biochemical mechanisms involved in enhanced gene expression is downstream of ERK phosphorylation. To elucidate such underlying mechanisms, this study presents a theoretical model of an anchored cell, representing an *in vitro* chondrocyte, in an ultrasound field. The model results showed that the mechanical energy storage is maximized at the chondrocyte’s resonant frequency and the energy density in the nucleus is almost twice as high as in the cytoplasm. Next, a mechanochemical model was developed to link the mechanical stimulation of ultrasound and the increased mechanical energy density in the nucleus to the downstream targets of the ERK pathway. This study showed for the first time that ultrasound stimulation induces frequency dependent gene expression as a result of altered rates of transcription factors binding to chromatin.

## Introduction

Over the past decades, low-intensity pulsed ultrasound (LIPUS) has been shown to induce bioeffects in tissue and promote bone fracture healing, and now therapeutic ultrasound devices are available on the market [1–4]. These successes drove researchers to study the effect of LIPUS on cartilage repair and restoration, even though the bioeffects were not completely understood [5]. Most published *in vivo* and *in vitro* cartilage restoration applications used LIPUS regimens at 1.0–1.5 MHz and intensities ranging from 1–50 mW/cm^2^ which were empirically derived from initial experiments with bone without accounting for cell and tissue properties, thus tending to variable results [6–12]. In order to optimize the regime for cartilage repair our group previously theoretically analyzed the response of suspended chondrocytes over a range of frequencies using continuous ultrasound and determined that they have a primary resonant frequency of 5.2 ± 0.8 MHz. Importantly, the study showed that cells in an ultrasound field act like any other oscillator—the mechanical energy couples most effectively when the stimulation occurs at resonance [11]. These findings were further extended to experimental validation using attached chondrocytes [11]. As transcriptional induction of load-inducible genes is independent from protein synthesis, they can act as a regulatory marker in the cellular response to external stimuli. Thus, the gene expression of selected load-inducible genes (c-fos, c-jun and c-myc) were monitored using a frequency of 5 MHz and compared to 2 and 8 MHz resulting in increased gene expression at the resonant frequency [11]. Additionally, previous experiments, using 5 MHz, also confirmed these findings and showed enhanced cellularity as well as increased matrix and protein synthesis at this resonant frequency [13–14]. However, the same experiments showed that extracellular regulated kinase (ERK) phosphorylation displayed no frequency dependency [11]. These experimental results coupled with the mathematical model developed by Louw et al. [11] suggests that the biochemical mechanisms involved in enhanced gene expression is downstream of ERK phosphorylation and that a particular nuclear mechanism is sensitive to the mechanical stimulation frequency [11]. However, what mechanisms involved are unknown. Louw et al. [11] posited that two possible mechanisms that could be effected are 1) nuclear transport and/or 2) chromatin binding.

Duvshani-Eshet et al. [15] experimentally studied the mechanisms involved in therapeutic ultrasound gene delivery. The effects observed in this study cannot be attributed to cavitation as the ultrasound intensity is below the cavitation threshold [16]. They monitored the intracellular trafficking of fluorescent plasmid—pGG during therapeutic ultrasound application. Their confocal studies showed that pGG was present in both the nucleus and cytoplasm with no increase in the nucleus observed post-therapeutic ultrasound application. Their data suggests that therapeutic ultrasound plays an important role in delivering DNA to the nucleus [15], thus affecting the mass flux into the nucleus. Krasovitski et al. [17] developed a theoretical cellular model capable of explaining the interaction mechanisms of ultrasound and tissue. Their model predicted that the bilayer cellular membrane is capable of transforming acoustic energy into mechanical stresses and strains at the subcellular level [17]. Their study supports the hypothesis that ultrasound stimulation has an effect on mass transfer across the nuclear membrane. Noriega et al. [18] studied the effects of ultrasound on chromatin remodeling in chondrocytes and fibroblasts. Using DAPI (4',6-diamidino-2-phenylindole) staining and differential scanning calorimetry techniques, they were able to show that ultrasound can induce chromatin remodeling [18]. These findings suggest that a potential mechanism involved in ultrasound induced bioeffects is enhanced binding of transcription factors to chromatin as a result of the flexing and bending of chromatin under ultrasound stimulation. These studies support the hypothesis that two downstream processes that could potentially explain enhanced transcription as a result of ultrasound stimulation at resonant frequencies are: (1) increased pERK transport to the nucleus and (2) increased binding rate of transcription factors to chromatin, such as ELK1, the transcription activator involved in the transcription of c-Fos. If either one or both mechanisms exhibit an optimum at the resonant frequency, it would explain the frequency-dependency of load-inducible gene expression.

This study proposes two theoretical models to elucidate the frequency sensitive mechanisms in low-intensity ultrasound enhanced gene expression. First, a cell attached to a plane, to mimic *in vitro* setups, is modeled to verify that the resonant frequency remains the same as that of a suspended cell and to determine the nuclear stored energy density versus frequency. Second, a kinetic model of the ERK signaling pathway is proposed and evaluated (using the stored energy density from the cell attached to a plane model as a parameter) to understand and predict the frequency dependent effect of ultrasound stimulation on the ERK signaling pathway, as phosphorylated extracellular regulated kinase (pERK) directly impacts the transcription of the load-inducible genes (c-Fos, c-Jun and c-Myc).

## Mathematical modeling

### Modeling cellular mechano-acoustics: Ultrasound interaction with an immobilized cell

To theoretically analyze the frequency sensitive mechanisms in ultrasound stimulation, the response of a chondrocyte attached to a plane to mimic *in vitro* setups was mathematically modeled. The presence of an immobilizing surface to which the chondrocytes can adhere to breaks the model symmetry and necessitates the use of a numerical method to solve for the spatial variation in ultrasonic stimulation. The numerical method used in this study was the finite element method, facilitated by COMSOL Multiphysics built-in Acoustics-Poroelastic Waves Interface (COMSOL Inc., Burlington, MA, USA).

The Poroelastic Waves Module solves the governing equations based on Biot’s theory [19–20] assuming a time-harmonic dependence, *p*(*x*, *t*) = *p*(*x*)*e*^*iωt*^ (as is the case in the application of continuous ultrasound stimulation). The governing equations are given by Eqs 1 and 2.

$$-\left(\rho_{av}-\frac{\rho_{f}^{2}}{\rho_{c}\left(\omega \right)}u\right)\omega^{2}u-\nabla \cdot \left(c:\varepsilon -\alpha_{B}p_{f}I\right)=\frac{\rho_{f}}{\rho_{c}\left(\omega \right)}\nabla p_{f}$$ (1)

$$\nabla \cdot \left(-\frac{1}{\rho_{c}}\left(\nabla p-\omega^{2}\rho_{f}u\right)\right)-\frac{k_{eq}^{2}p}{\rho_{c}}=\omega^{2}\alpha_{B}\nabla \cdot u$$ (2)

*ρ*_*av*_ is the average density defined by Eq 3, *ρ*_*c*_, defined by Eq 4, is the complex density which accounts for tortuosity, porosity and fluid density and *k*_*eq*_ is the wavenumber defined by Eq 5, *ρ*_*d*_ is the drained density of the porous material, *ρ*_*f*_ is the fluid density, **u** is the displacement vector, *ω* is angular frequency, ***c*** is the elasticity tensor, *ε* is the strain tensor, *α*_*B*_ is the Biot-Willis coefficient and *p* is pressure.

$$\rho_{av}=\rho_{d}+\epsilon_{P}\rho_{f}$$ (3)

$$\rho_{c}=\frac{\tau_{\infty }\rho_{f}}{\epsilon_{P}}+\frac{\mu_{f}}{i\omega k_{P}}$$ (4)

$$k_{eq}^{2}=\left(\epsilon_{P}\chi_{f}+\frac{\alpha_{B}-\epsilon_{P}}{K_{d}}\left(1-\alpha_{B}\right)\right)\omega^{2}\rho_{c}$$ (5)

*ϵ*_*P*_ is porosity, *τ*_∞_ is tortuosity, *k*_*p*_ is permeability, *μ*_*f*_ is the fluid viscosity, *χ*_*f*_ is the fluid compressibility and *K*_*d*_ is the bulk modulus. The values were obtained from Louw et al. [11] and listed in Table 1.

**Table 1:** Material properties used in the Biot theory.

Cytoplasm			
Bulk Medium			
Bulk Modulus (Pa)	*K*_*d*_	500	[11]
Poisson's Ratio	*ν*	0.38	[21]
Bulk Density (kg/m^3^)	*ρ*_*d*_	300	[22]
Permeability (m^2^)	*k*_*p*_	7*10^−19^	$\mu_{f}/\bar{\omega }$ [23]
Porosity	*є*_*p*_	0.75	[22]
Biot-Willis Coefficient	*α*_*B*_	0.9999	*1-K*_*d*_*/K*_*s*_
Tortuosity Factor	*τ*_*∞*_	1.2	[24]
Fluid Phase			
Density (kg/m^3^)	*ρ*_*f*_	992.52	[25]
Vicosity (Pa∙s)	*μ*_*f*_	0.7*10^−3^	[26]
Compressibility (1/Pa)	χ_*f*_	4.35*10^−10^	*1/K*_*f*_
Nucleus			
Bulk Medium			
Bulk Modulus (Pa)	*K*_*d*_	2*10^3^	[27]
Poisson's Ratio	*ν*	0.38	[11]
Bulk Density (kg/m^3^)	*ρ*_*d*_	400	[11]
Permeability (m^2^)	*k*_*p*_	7*10^−19^	$\mu_{f}/\bar{\omega }$ [23]
Porosity	*є*_*p*_	0.65	[28]
Biot-Willis Coefficient	*α*_*B*_	0.9996	*1-K*_*d*_*/K*_*s*_
Tortuosity Factor	*τ*_*∞*_	2	[24]
Fluid Phase			
*See cytoplasm fluid phase*			

**Fig 1:**
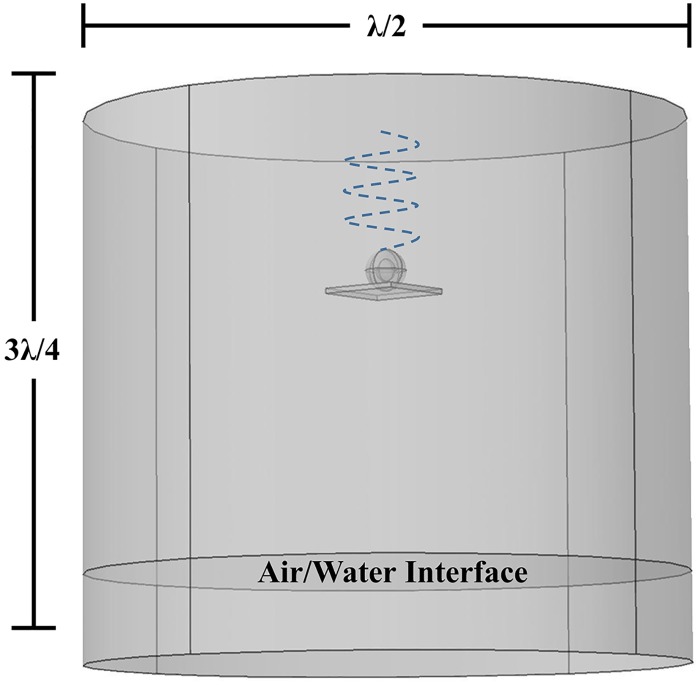
Model geometry. Cell attached to a planar surface (polystyrene to mimic the properties of a 6-welled plate) immersed in fluid (growth media). The transducer (ultrasound source) is positioned at the top. The cell position changes so that it always lies at an antinode or node.

To identify the resonant frequency, the cell was represented as a set of four concentric spheres representing the nucleus, nuclear envelope, cytoplasm and cellular membrane. The experimental model was bovine chondrocytes, with cell and nuclear radii of 6.5 and 3.5 μm [27, 29], respectively, and thicknesses of the plasma membrane and nuclear envelope of 15 and 40 nm, respectively [30]. Each of the four cellular domains are most appropriately modeled as biphasic media [21, 27, 31, 32–34]. The geometry mimics typical *in vitro* experimental setups. Here the cell is attached to a planar surface immersed in a cylinder (well) filled with growth media. The dimensions of the surface was 2*cellular radius x 2*cellular radius x cellular radius/2. The pressure wave source (ultrasound) is positioned above the plate as shown in Fig 1 with an amplitude of 14 kPa which was the same amplitude used in Louw et al. [11]. The position of the planar surface varies per frequency to ensure the location of the cell is either at an antinode or node. This ensures the pressure amplitude at the chondrocyte’s position remains the same for all frequencies, allowing a direct comparison. Water properties are used for the growth media and polystyrene as the planar surface, to replicate the properties of well plates used in experimental setups. Two boundary conditions were examined in this study, using a sound hard boundary layer which assumes the normal component of acceleration is zero and cylindrical wave radiation which allows the outgoing wave to leave with minimal reflection [20].

Acoustic problems have wavelike solutions that are characterized by the wavelength, *λ*, where *λ = c/f* (*c* is the speed of sound and *f* is frequency), which needs to be resolved by the mesh size. To realistically model these problems there needs to be ten to twelve degrees of freedom per wavelength [20]. Thus, when determining the stored energy density, to minimize the degrees of freedom and computational cost, the geometry was reduced to a height of 3*λ*/4 and a width of *λ*/2 and the cell was treated as two concentric spheres representing the nucleus and cytoplasm. However, trials were ran with larger dimensions to study the geometry dimension effects on the resonant frequency. Additionally, a mesh optimization study was conducted to ensure the resonant frequency did not shift and the mesh size was appropriate. It is important to note that a linear model was used in this study which would predict a peak that approaches infinity at the resonant frequency. The geometry dimensions ensured the formation of a standing wave as a result of the air/water interface. This is also seen in *in vitro* experimental setups as a result of the air/polystyrene interface when the transducer is positioned at the top. A tetrahedral element was used to mesh the geometry resulting in 40,000–50,000 elements (varies per frequency) and solved using an Intel Core i5 desktop computer with 16 GB RAM. A frequency dependent analysis was conducted and approximate solution time was 1–5 hours.

### Modeling frequency dependence of load-inducible gene expression

The final aim of the study is to predict the ERK signaling pathway mechanisms involved in frequency dependent gene expression. This will begin to unravel the question of how the cell utilizes the mechanical energy absorbed from ultrasound application. Experimental studies have shown that mechanical stimulation, including ultrasound, initiates the well-studied mitogen activated protein kinase (MAPK) pathway and that gene and protein expression vary depending on the frequency of the ultrasound applied [35]. However, the mechanisms within the signaling pathway affected by frequency is unknown. Louw et al. [11] shows that the mechanism is downstream of ERK phosphorylation and posited that two possible mechanisms could be involved: 1) nuclear transport and/or 2) chromatin binding. This study focuses on these two mechanisms, introduces frequency dependency at these points within the model and analyzes the downstream effects on one of the load inducible genes, c-Fos.

**Fig 2:**
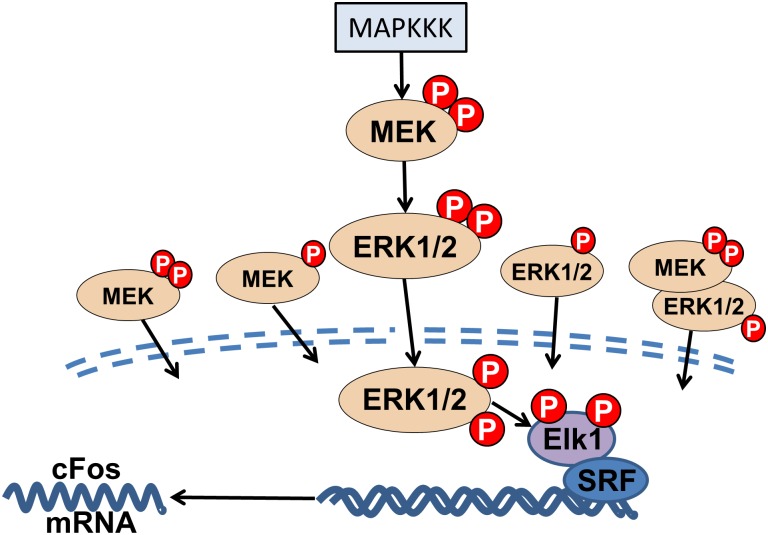
Segment of ERK pathway. Ultrasound leads to the phosphorylation/activation of an unknown enzyme (MAPKKK) which kick starts the ERK signaling pathway resulting in gene expression.

Whitney et al. [35] showed that ultrasound activates the ERK using multiple frequencies. Thus, to analyze the frequency dependent mechanisms, the ERK pathway beginning with mitogen-activated protein kinase kinase kinase (MAPKKK) and ending with c-Fos gene expression is modeled using the approach described in Huang and Ferrell [36] and Harrington et al. [37]. The Huang and Ferrell [36] model focuses on the pathway beginning with a stimulus (unknown enzyme) activating MAPKKK, which initiates the pathway, and ends with ERK phosphorylation. The specific MAPKKK involved in ultrasound initiation of the ERK cascade is unknown, however, a MAPKKK is always involved in the cascade. In this model, we start with the stimulus, which resembles an on/off switch, activating MAPKKK. For our purposes, we treat the stimulus as on when ultrasound is applied and off when ultrasound is turned off. The methods described in Harrington et al. [37] extend this model by incorporating nuclear transport of ERK, Mitogen-Activated Protein Kinase/ERK Kinase (MEK) and ERK-MEK complexes. The pathway is terminated with chromatin binding and gene expression using Eqs 6–8. This links nuclear transported pERK with c-Fos gene expression. pERK induces c-Fos gene expression through ELK1, a transcription activator, and serum response factor (SRF), a transcription factor already bounded to chromatin. pERK phosphorylates ELK1, followed by ELK1 binding to SRF and inducing gene expression. The modeled pathway is shown in Fig 2. Note the use of the subscript “n” to indicate species inside the nucleus.

$$ERKPPn+ELK1\overset{}{↔}ERKPP-ELK1\overset{}{\to }ERKPn+ELK1P$$ (6)

$$ERKPPn+ELK1P\overset{\;}{↔}ERKPP-ELK1P\overset{\;}{\to }ERKPn+ELK1PP$$ (7)

$$SRF+ELK1PP\overset{}{↔}SRF-ELK1PP\overset{\;}{\to }SRF-ELK1PP+cFos$$ (8)

Combining all equations results in a system of 34 differential equations (see the supplemental information for all equations). The system was solved using MATLAB R2015a’s built in function ODE45 with a solution time of approximately five minutes and a time step of one sec. The initial conditions for all species concentrations were set to zero except the stimulus which had a concentration of 0.05 μM while the ultrasound source was on and zero when the source was removed. In order to compare values to experimental data reported in Louw et al. [11], the length of time the ultrasound was applied was inversely proportional to frequency to ensure the number of cycles remained constant (3x10^8^ cycles) [11]. Thus, the time the stimulus was on was inversely proportional to frequency. Louw et al. [11] determined that the peak c-Fos concentration was calculated at approximately 1.5 hours, therefore the total model runtime replicated this timeframe and the c-Fos concentration at the end of this time was reported.

The experimentally observed frequency dependent gene expression was incorporated into the mathematical model using a phenomenological approach for nuclear transport and/or chromatin binding rate. Both the driving force for nuclear transport and the chromatin binding rate can be related to the mechanical energy density within the nucleus, *U*, using Eq 9. The mechanical energy density is the energy stored by the cell undergoing deformation as a result of the ultrasound field. It is the cycle average of the elastic energy, obtained by Eq 9.

$$U=\frac{1}{T}\int_{0}^{T}\frac{1}{2}\sigma \left(t\right):\varepsilon \left(t\right)dt$$ (9)

*T* is the period and ***σ*(*t*)** is the stress tensor. The mechanical energy density is in turn related to the frequency of the ultrasound stimulation, as discussed above.

#### Frequency dependence of nuclear transport

The pathway involves the transport of ERK1/2PP, ERK1/2P, ERK1/2, MEKPP, ERK1/2-MEKPP, and ERK1/2P-MEKPP complexes across the nuclear envelope (Eqs 10–16). The import and export rate constants, representing the cytoplasm/nucleus and nucleus/cytoplasm transport rates respectively, were obtained from Harrington et al. [37].

$$ERK\overset{\;}{↔}ERK_{n}$$ (10)

$$ERKP\overset{\;}{↔}ERKP_{n}$$ (11)

$$ERKPP\overset{\;}{↔}ERKPP_{n}$$ (12)

$$MEKP\overset{\;}{↔}MEKP_{n}$$ (13)

$$MEKPP\overset{\;}{↔}MEKPP_{n}$$ (14)

$$ERK-MEKPP\overset{\;}{↔}ERK-MEKPP_{n}$$ (15)

$$ERKP-MEKPP\overset{\;}{↔}ERKP-MEKPP_{n}$$ (16)

ERK can translocate from the cytoplasm to the nucleus by passive transport. Chemical potential gradients are the fundamentally correct driving forces of diffusion [38], thus we model frequency dependent passive transport using Fick’s law in terms of chemical potential, Eqs 17 and 18, which relates chemical potential, *μ*, to concentration (in an ideal solution) [39].

$$\text{Molar Flux}=\text{Rate of transport}=-Dc_{t}\nabla x_{i}=-\frac{Dc_{t}x_{i}}{RT}\frac{\partial \mu_{i}}{\partial z}$$ (17)

where

$$\mu_{i}=\mu_{i}^{o}+RT\text{ln}c_{i}$$ (18)

Substituting Eqs 18 into 17 results in Eq 19.

$$\begin{array}{ll} \text{Rate of transport} & =-\frac{Dc_{i}}{RT}\left(\frac{\partial \mu_{i}^{o}}{\partial z}+\frac{RT}{c_{i}}\frac{\partial c_{i}}{\partial z}\right)=-D\left(\frac{c_{i}}{RT}\frac{\partial \mu_{i}^{o}}{\partial z}+\frac{\partial c_{i}}{\partial z}\right)\\ & \approx -k\left(\frac{\bar{c}}{RT}\;\left(\mu_{nucleus}^{o}-\mu_{cytoplasm}^{o}\right)+\left({\bar{c}}_{nucleus}-{\bar{c}}_{cytoplasm}\right)\right) \end{array}$$ (19)

$\frac{\partial \mu_{i}^{o}}{\partial z}$ is the difference between the chemical potential reference state of species *i* in the nucleus and cytoplasm, $\bar{c}$ is the average concentration of the molecule in the nucleus and cytoplasm and *k* is the rate constant defined in Harrington et al. [37]. Assuming that the Gibbs free energy of a molecule varies proportionally to the stored mechanical energy density *U* in the system such that *μ* = *α*_*μ*_*U*, the rate of transport between the nucleus and the cytoplasm can be related to *U* by Eq 20. Recall that the mechanical energy density *U* is influenced by the ultrasonic stimulation.

$$\text{Rate of transport}=-k\left(\frac{\alpha_{\mu }\bar{c}}{RT}\;\left(U_{nucleus}-U_{cytoplasm}\right)+\left({\bar{c}}_{nucleus}-{\bar{c}}_{cytoplasm}\right)\right)$$ (20)

Where parameter *α*_*μ*_ is a proportionality constant (to be determined) between the partial molar Gibbs free energy and the frequency dependent energy density *U*. The values of *U* were obtained from the immobilized cell model.

#### Frequency dependence of chromatin binding

Frequency dependent chromatin binding is incorporated into the modeled pathway by relating the change in internal energy to the rate constants in Eq 21 using Arrhenius Law.

$$ELK1PP+SRF\overset{k_{B1},k_{-B1}}{↔}ELK1PP\cdot SRF\overset{k_{B2}}{\to }ELK1PP\cdot SRF+cFos$$ (21)

The binding rates as a function of frequency are calculated using Eq 22.

$$k_{B1}=Ae^{\frac{-E_{a}}{RT}}=Ae^{\frac{-\left(E_{a0}-\alpha_{E}U\right)}{RT}}=Ae^{\frac{-E_{a0}}{RT}}e^{\frac{\alpha_{E}U}{RT}}=k_{B1_{0}}e^{\frac{\alpha_{E}U}{RT}}$$ (22)

*E*_*a*0_ is the initial activation energy, *α*_*E*_ is the proportionality constant relating the decrease in the activation energy to frequency dependent energy density, *U*, and $k_{B1_{0}}=Ae^{\frac{-E_{a0}}{RT}}$.

## Results and discussion

### Cells adhered to planar surfaces

**Fig 3:**
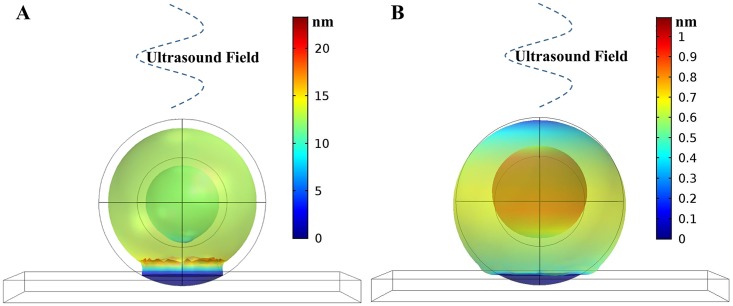
Model of cell attached to a plane (90° with respect to the incident planar ultrasonic wave), the color represents displacement in nanometers. A) Cell at anti-node in 5 MHz field. B) Cell at node in 8 MHz field.

Fig 3 provides a visual depiction of the effects on a cell in an ultrasound field. Fig 3A, shows a cell attached to a plane and located at a pressure anti-node in a 5 MHz ultrasound field. The cell expands and contracts primarily in the radial direction, creating an alternating tensile/compressive force on the adhesion points normal to the plane. The cell pulls away from the adhesion point (the displacements are enlarged for clarity). In Fig 3B, the cell is located at a pressure node in an 8 MHz ultrasound field. A pressure node coincides with a velocity antinode, resulting in the water velocity oscillating at maximum amplitude. This alternating flow exerts a rolling action on the cell, thence the adhesion points experience stronger forces parallel to the plane (shear).

**Fig 4:**
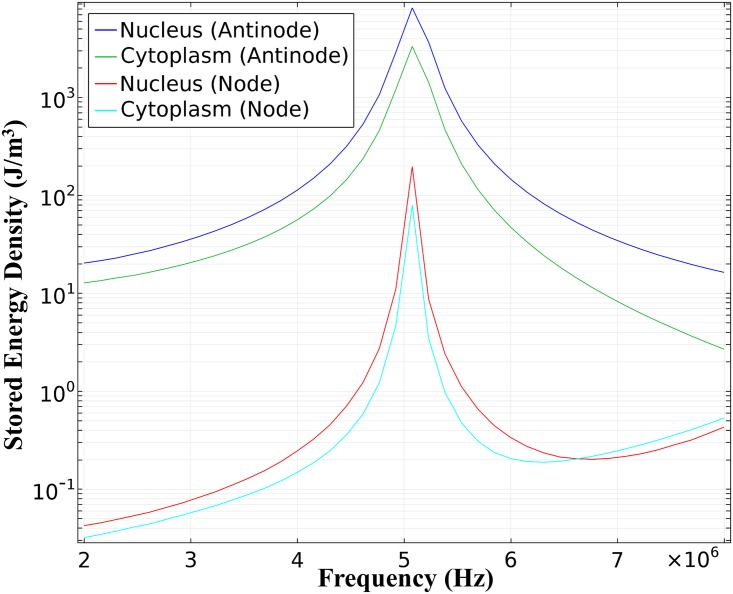
Total mechanical energy density. Total mechanical energy density in the cytoplasm and nucleus for cells attached to a plane located at a pressure anti-node and node.

The total mechanical energy density *U* stored in the cell (elastic and kinetic) as a function of frequency was calculated and shown in Fig 4. The mechanical energy density in the nucleus is roughly double the amount in the cytoplasm, because the nucleus tends to be 3 to 4 times stiffer than the surrounding cytoplasm in chondrocytes [27]. The position of the planar surface was varied to analyze the difference in the stored mechanical energy density versus the pressure wave (ultrasound) incidence angle; there was no notable difference. The stored mechanical energy density in the cytoplasm and nucleus for a cell located at a pressure anti-node and pressure node are overlaid in the plot. Comparing the pressure anti-node position with the cell located at a pressure node, the width of the plots are broader, thus mechanical energy is stored over a wider frequency range when cells are located at pressure anti-nodes. However, the cell position in an ultrasound field applied to the knee joint is unknown which explains the importance of primary resonance. As seen in the plot, the primary resonance is approximately 5.1 MHz, a slight shift from 5.2 MHz for freely-suspended cells calculated in Louw et al. [11]. Although this study utilized a linear model where the energy coupled to the cell approaches infinity at the resonant frequency, one can conclude that the energy is dependent on the intensity received at the defect site. The higher the intensity the higher the energy coupled to the cell. For further details of nonlinear effects on the resonant frequency and energy coupled to the cell the reader is referred to Miller et al. [40]. The same effects were observed when varying the boundary conditions. The amount of energy coupled to the cell increased using a sound hard versus a cylindrical wave radiation boundary condition, however, the resonant frequency did not change.

### Frequency dependent cell signaling

Biochemical pathways are altered by the dilatation and shear effects on cells. Published data revealed frequency dependency downstream of ERK phosphorylation [11]. Thus, we analyzed 1) increased transport rates in and out of the nucleus and 2) increased binding rates of transcription factors to the chromatin as possible mechanotransduction mechanisms.

**Fig 5:**
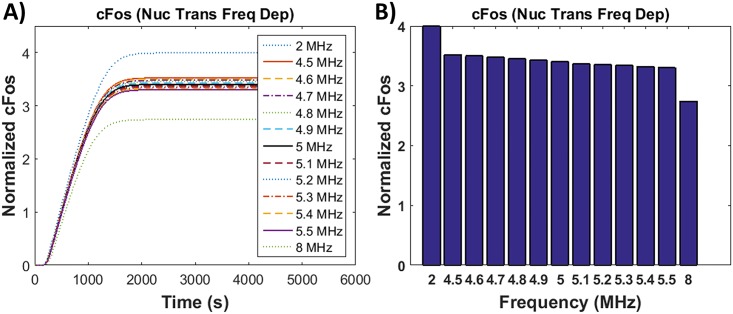
c-Fos gene expression with modeled frequency sensitive nuclear transport using Eq 20. A) c-Fos gene expression versus time for frequency dependent nuclear transport, B) c-Fos gene expression at a snapshot in time, 1.5 hours following the completion of ultrasound stimulation.

Frequency dependent nuclear transport was incorporated in the modeled pathway using Eq 20 and the stored energy density from Fig 4. The c-Fos (mRNA) expression profile was studied over a range of frequencies from 1 to 9 MHz and the values for frequencies 2, 8 and the resonance bandwidth frequencies (4.5–5.5 MHz) are shown in Fig 5A. A snapshot of the c-Fos concentration at 1.5 hrs are shown in Fig 5B. The resulting concentrations were normalized to the concentration obtained experimentally using 5 MHz ultrasound stimulation. As seen, the amount of c-Fos decreases as the frequency increases. This would be observed if molecules are being exported out of the nucleus faster than the chromatin binding rate. This suggests that nuclear transport is not a mechanism behind the frequency dependent gene expression results seen in experiments.

**Fig 6:**
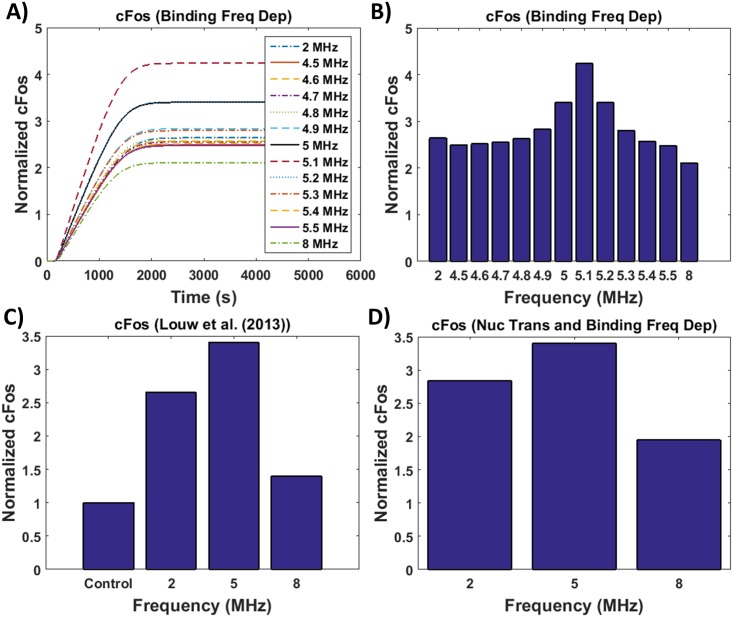
c-Fos gene expression with modeled frequency sensitive chromatin binding using Eq 22. A) c-Fos gene expression versus time with frequency sensitive chromatin binding, B) c-Fos gene expression at 1.5 hours following the completion of ultrasound stimulations, C) experimental results from Louw et al. [11] and D) c-Fos gene expression at 1.5 hours following the completion of ultrasound stimulation for modeled frequency sensitivity for both nuclear transport and chromatin binding.

To test the chromatin binding hypothesis, the stored energy density and Eq 22 was used to calculate c-Fos gene expression for frequency dependent nuclear binding, analyzing a frequency sweep from 1 to 9 MHz. Results are shown in Fig 6A. A snapshot at 1.5 hours shown in Fig 6B are comparable to experimental results (Fig 6C). Fig 6B shows a higher concentration at 2 than 4 MHz even though Fig 4 shows slightly higher stored energy density at 4 than 2 MHz, this is attributed to the length of the stimulation period. To maintain the same number of cycles, to replicate experimental procedures [11] as discussed previously, stimulation time is decreased as frequency is increased. These results suggest that chromatin binding is a mechanism involved in frequency dependent gene expression. Combining both postulates, frequency dependent nuclear transport and chromatin binding, also results in frequency dependent gene expression (Fig 6D). The kinetic rate constants for the portion of the modeled pathway beginning with pERK-ELK1 binding are unknown parameters, to accommodate for this, a range of rate constants were examined in this study. The c-Fos gene expression profile pattern remained the same over the entire range, frequency independent with frequency dependent nuclear transport and frequency dependent with frequency dependent chromatin binding. The results from relating the increase in internal energy to binding and transport rates offer an explanation on how the cell utilizes the energy absorbed from ultrasound.

The cellular models of a cell attached to a plane and of a suspended cell predicted that the resonant frequency is approximately 5 MHz. However, an *in vivo* cell is embedded in the pericellular matrix (PCM), which is involved in the biomechanical response of the cell, and surrounded by the extracellular matrix (ECM), with vastly different mechanical properties. Thus, chondrocyte’s biomechanical environment could cause a shift in the resonant frequency. Hence, future work should include the PCM and ECM to determine the *in vivo* chondrocyte’s resonant frequency. Additional future work should also include the development of a system to obtain real time cytoplasm to nucleus shuttling and chromatin binding data while ultrasound is applied which will enable the validation of this theoretical work. Finally, this study focused on continuous ultrasound versus pulsed ultrasound, however, the resonant frequency will not change but the energy coupled to the cell will be decreased with pulsed ultrasound as a result of the duty cycle which allows the cell to return to the rest phase.

## Conclusions

In summary, the resonant frequencies of anchored chondrocytes in a standing ultrasound field were calculated and the stored energy density determined. To link the resonance study with the gene expression findings, we explored intracellular processes that could be affected by ultrasound stimulated pathways that lead to pERK and gene transcription. We posit that higher mechanical energy density in the nucleus at resonance conditions increases chromatin strain and consequently enhances the binding rate of ELK1 to SRF [18]. The model suggests that mechanical stimulation induces frequency dependent gene expression as a result of altered chromatin binding rates, not altered nuclear transport. This postulate offers an explanation of our earlier *in vitro* studies that showed that optimal expression of c-Fos, c-Jun and c-Myc occurred when cells were mechanically excited at the resonant frequency [11].

## Supporting information

S1 File Pathway supplement. System of equations required to solve for the frequency dependent mechanism.
(DOCX)
