# Supplementary material for: Frequency sensitive mechanism in low-intensity ultrasound enhanced bioeffects
Source: PLoS One. 2017 Aug 1;12(8):e0181717. doi: 10.1371/journal.pone.0181717 (PMC5538718; doi:10.1371/journal.pone.0181717)
Supplement: S1 File — System of equations required to solve for the frequency dependent mechanism. (DOCX) [file pone.0181717.s001.docx]

# Pathway supplement

The ERK Pathway was modeled based on the following reactions:

## MAPK pathway

| Reaction | Reference |
| --- | --- |
| $\boldsymbol{Stimulant}+MAPKKK\underset{\to}{k}\boldsymbol{MAPKKK}$ | [1] |
| $\boldsymbol{MAPKKK}+MEK\underset{\leftrightarrow}{f_{1},b_{1}}MEK-\boldsymbol{MAPKKK}\underset{\to}{k_{1}}MEKP+MAPKKK$ | [1] |
| $\boldsymbol{MAPKKK}+MEKP\underset{\leftrightarrow}{f_{2},b_{2}}MEKP-\boldsymbol{MAPKKK}\underset{\to}{k_{2}}MEKPP+MAPKKK$ | [1] |
| $MEKPP+ERK\underset{\leftrightarrow}{f_{3},b_{3}}ERK-MEKPP\underset{\to}{k_{3}}ERKP+MEKPP$ | [1] |
| $MEKPP+ERKP\underset{\leftrightarrow}{f_{4},b_{4}}ERKP-MEKPP\underset{\to}{k_{4}}ERKPP+MEKPP$ | [1] |

## Deactivation

| Reaction | Reference |
| --- | --- |
| $\boldsymbol{MAPKKK}+PP\underset{\to}{kd_{1}}MAPKKK$ | [1, 2] |
| $MEKPP+PP\underset{\to}{kd_{2}}MEKP$ | [1, 2] |
| $MEKP+PP\underset{\to}{kd_{3}}MEK$ | [1, 2] |
| $ERKPP+PP\underset{\to}{kd_{4}}ERKP$ | [1, 2] |
| $ERKP+PP\underset{\to}{kd_{5}}ERK$ | [1, 2] |

## Nuclear translocation

| Reaction | Reference |
| --- | --- |
| $ERK\underset{\leftrightarrow}{N{Ti}_{1}, NTe_{1}}ERK_{n}$ | [3, 4] |
| $ERKP\underset{\leftrightarrow}{N{Ti}_{2}, NTe_{2}}ERKP_{n}$ | [3, 4] |
| $ERKPP\underset{\leftrightarrow}{N{Ti}_{3}, NTe_{3}}ERKPP_{n}$ | [3, 4] |
| $MEK\underset{\leftrightarrow}{N{Ti}_{4}, NTe_{4}}MEKP_{n}$ | [3, 4] |
| $MEKP\underset{\leftrightarrow}{N{Ti}_{5}, NTe_{5}}MEKP_{n}$ | [3, 4] |
| $MEKPP\underset{\leftrightarrow}{N{Ti}_{6}, NTe_{6}}MEKPP_{n}$ | [3, 4] |
| $ERK-MEKPP\underset{\leftrightarrow}{N{Ti}_{7}, NTe_{7}}ERK-MEKPP_{n}$ | [3, 4] |
| $ERKP-MEKPP\underset{\leftrightarrow}{N{Ti}_{8}, NTe_{8}}ERKP-MEKPP_{n}$ | [3, 4] |

## MAPK pathway in nucleus

| Reaction | Reference |
| --- | --- |
| $MEKPP_{n}+ERK_{n}\underset{\leftrightarrow}{f_{5},b_{5}}ERK-MEKPP_{n}\underset{\to}{k_{5}}ERKP_{n}+MEKPP_{n}$ | [3] |
| $MEKPP_{n}+ERKP_{n}\underset{\leftrightarrow}{f_{6},b_{6}}ERKP-MEKPP_{n}\underset{\to}{k_{6}}ERKPP_{n}+MEKPP_{n}$ | [3] |
| $MEKPP_{n}+PP_{n}\underset{\to}{kd_{8}}MEKP_{n}$ | [2] |
| $MEKP_{n}+PP_{n}\underset{\to}{kd_{9}}MEK_{n}$ | [2] |
| $ERKPP_{n}+PP_{n}{\underset{\to}{kd_{10}}ERKP}_{n}$ | [2] |
| $ERKP_{n}+PP_{n}\underset{\to}{kd_{11}}ERK_{n}$ | [2] |

## ELK1 phosphorylation and chromatin binding

| Reaction | Reference |
| --- | --- |
| $ERKPPn+ELK1\underset{\leftrightarrow}{f_{7},b_{7}}ERKPP-ELK1\underset{\to}{k_{7}}ERKPPn+ELK1P$ | [5-7] |
| $ERKPPn+ELK1P\underset{\leftrightarrow}{f_{8},b_{8}}ERKPP-ELK1P\underset{\to}{k_{8}}ERKPPn+ELK1PP$ | [5-7] |
| $SRF+ELK1PP\underset{\leftrightarrow}{f_{9},b_{9}}SRF-ELK1PP\underset{\to}{k_{9}}SRF-ELK1PP+cFos$ | [8, 9] |
| $ELK1PP-SRF+mSin3A\underset{\to}{k_{10}}ELK1PP+SRF+mSin3A$ | [8] |
| $ELK1P+PP\underset{\to}{{kd}_{13}}ELK1+PP$ | [8, 10] |
| $ELK1PP+PP\underset{\to}{{kd}_{12}}ELK1P+PP$ | [8, 10] |
| $ELK1PPSRF+PP\underset{\to}{{kd}_{14}}ELK1P+SRF+PP$ | [8, 10] |

Note: ERK stands for either ERK1 or ERK2. Bold represents activated species.

The pathway was modeled using MATLAB R2015a and the following differential algebraic equation system consisting of 34 ordinary differential equations and five algebraic equations.

## Ordinary differential equations

The reactions above give rise to the following rate equations [1, 3]:

$$\frac{d\left[ MEK\boldsymbol{MAPKKK} \right]}{dt}=f_{1}[MEK][\boldsymbol{MAPKKK}\boldsymbol{]}-\left( b_{1}+k_{1} \right)[MEK\boldsymbol{MAPKKK}]$$

$$\frac{d\left[ MEKP \right]}{dt}=k_{1}[MEK\boldsymbol{MAPKKK}]-f_{2}[MEKP][\boldsymbol{MAPKKK}]+b_{2}[MEKP\boldsymbol{MAPKKK}]-kd_{3}[MEKP][KKPase]+kd_{2}[MEKPP][KKPase]$$

$$\frac{d\left[ \boldsymbol{MAPKKK} \right]}{dt}=k*Stimulant-kd_{1}[\boldsymbol{MAPKKK}]-f_{1}[MEK][\boldsymbol{MAPKKK}]+(b_{1}+k_{1})[MEK\boldsymbol{MAPKKK}]-f_{2}[MEKP][\boldsymbol{MAPKKK}]+(b_{2}+k_{2})[MEKP\boldsymbol{MAPKKK}]$$

$$\frac{d\left[ MEKPP \right]}{dt}=k_{2}\left[ MEKP\boldsymbol{MAPKKK} \right]-f_{3}\left[ ERK1 \right]\left[ MEKPP \right]+(b_{3}+k_{3})[MEKPPERK1]-f_{3}[ERK2][MEKPP]+(b_{3}+k_{3})[MEKPPERK2]-kd_{2}[MEKPP][KKPase]-f_{4}[ERK2P][MEKPP]+(b_{4}+k_{4})[MEKPPERK2P]-f_{4}[ERK1P][MEKPP]+(b_{4}+k_{4})[MEKPPERK1P]-NTi_{6}[MEKPP]+NTe_{6}[MEKPP_{n}]$$

$$\frac{d\left[ MEKPPERK1 \right]}{dt}=f_{3}\left[ ERK1 \right]\left[ MEKPP \right]-\left( b_{3}+k_{3} \right)\left[ MEKPPERK1 \right]-NTi_{7}\left[ MEKPPERK1 \right]+NTe_{7}\left[ MEKPPERK1_{n} \right]$$

$$\frac{d\left[ ERK1P \right]}{dt}=k_{3}[MEKPPERK1]-f_{4}[ERK1P][MEKPP]+b_{4}[MEKPPERK1P]-NTi_{2}[ERK1P]+NTe_{2}[ERK1P_{n}]+kd_{4}[ERK1PP][KPase]-kd_{5}[ERK1P][KPase]$$

$$\frac{d\left[ MEKPPERK2 \right]}{dt}=f_{3}\left[ ERK2 \right][MEKPP]-(b_{3}+k_{3})[MEKPPERK2]-NTi_{7}[MEKPPERK2]+NTe_{7}[MEKPPERK2_{n}]$$

$$\frac{d\left[ ERK2P \right]}{dt}=k_{3}[MEKPPERK2]-f_{4}[ERK2P][MEKPP]+b_{4}[MEKPPERK2P]-NTi_{2}[ERK2P]+NTe_{2}[ERK2P_{n}]+kd_{4}[ERK2PP][KPase]-kd_{5}[ERK2P][KPase]$$

$$\frac{d\left[ MEKPPERK1P \right]}{dt}=f_{4}\left[ ERK1P \right][MEKPP]-(b_{4}+k_{4})[MEKPPERK1P]-NTi_{8}[MEKPPERK1P]+NTe_{8}[MEKPPnERK1P_{n}]$$

$$\frac{d\left[ ERK1PP \right]}{dt}=k_{4}[MEKPPERK1P]-NTi_{3}[ERK1PP]+NTe_{3}[ERK1PP_{n}]-kd_{4}[ERK1PP][KPase]$$

$$\frac{d\left[ MEKPPERK2P \right]}{dt}=f_{4}\left[ ERK2P \right][MEKPP]-(b_{4}+k_{4})[MEKPPERK2P]-NTi_{8}[MEKPPERK2P]+NTe_{8}[MEKPPERK2P_{n}]$$

$$\frac{d\left[ ERK2PP \right]}{dt}=k_{4}[MEKPPERK2P]-NTi_{3}[ERK2PP]+NTe_{3}[ERK2PP_{n}]-kd_{4}[ERK2PP][KPase]$$

$$\frac{d\left[ ERK1_{n} \right]}{dt}=NTi_{1}[ERK1]-NTe_{1}[ERK1_{n}]-f_{5}[ERK1_{n}][MEKPP_{n}]+b_{5}[MEKPPERK1_{n}]+kd_{11}[ERK1P_{n}][KPase_{n}]$$

$$\frac{d\left[ ERK2_{n} \right]}{dt}=NTi_{1}[ERK2]-NTe_{1}[ERK2_{n}]-f_{5}[ERK2_{n}][MEKPP_{n}]+b_{5}[MEKPPERK2_{n}]+kd_{11}[ERK2P_{n}][KPase_{n}]$$

$$\frac{d\left[ ERK1P_{n} \right]}{dt}=NTi_{2}\left[ ERK1P \right]-NTe_{2}\left[ ERK1P_{n} \right]+k_{5}\left[ MEKPPERK1_{n} \right]-f_{6}\left[ ERK1P_{n} \right]\left[ MEKPP_{n} \right]+b_{6}\left[ MEKPPERK1P_{n} \right]+kd_{10}[ERK1PP_{n}][KPase_{n}]-kd_{11}[ERK1P_{n}][KPase_{n}]$$

$$\frac{d\left[ ERK2Pn \right]}{dt}=NTi_{2}[ERK2P]-NTe_{2}[ERK2P_{n}]+k_{5}[MEKPPERK2_{n}]-f_{6}[ERK2P_{n}][MEKPP_{n}]+b_{6}[MEKPPERK2P_{n}]+kd_{10}[ERK2PP_{n}][KPase_{n}]-kd_{11}[ERK2P_{n}][KPase_{n}]$$

$$\frac{d\left[ ERK1PP_{n} \right]}{dt}=NTi_{3}[ERK1PP]-NTe_{3}[ERK1PP_{n}]+k_{6}[MEKPPERK1P_{n}]-kd_{10}[ERK1PP_{n}][KPase_{n}]-f_{7}[ERK1PP_{n}][ELK1]+(b_{7}+k_{7})[ERK1PPELK1_{n}]-f_{8}[ERK1PP_{n}][ELK1P]+(b_{8}+k_{8})[ERK1PPELK1P_{n}]$$

$$\frac{d\left[ ERK2PP_{n} \right]}{dt}=NTi_{3}[ERK2PP]-NTe_{3}[ERK2PP_{n}]+k_{6}[MEKPPERK2P_{n}]-kd_{10}[ERK2PP_{n}][KPase_{n}]-f_{7}[ERK2PP_{n}][ELK1]+(b_{7}+k_{7})[ERK2PPELK1_{n}]-f_{8}[ERK2PP_{n}][ELK1P]+(b_{8}+k_{8})[ERK2PPELK1P_{n}]$$

$$\frac{d\left[ MEK_{n} \right]}{dt}=NTi_{4}[MEK]-NTe_{4}[MEK_{n}]+kd_{9}[MEKP_{n}][KKPase_{n}]$$

$$\frac{d\left[ MEKP_{n} \right]}{dt}=NTi_{5}[MEKP]-NTe_{5}[MEKP_{n}]-kd_{9}[MEKP_{n}][KKPase_{n}]+kd_{8}[MEKPP_{n}][KKPase_{n}]$$

$$\frac{d\left[ MEKPP_{n} \right]}{dt}=NTi_{6}[MEKPP]-NTe_{6}[MEKPP_{n}]-f_{5}[ERK1_{n}][MEKPP_{n}]+(b_{5}+k_{5})[MEKPPERK1_{n}]-f_{5}[ERK2_{n}][MEKPP_{n}]+(b_{5}+k_{5})[MEKPPERK2_{n}]-f_{6}[ERK1P_{n}][MEKPP_{n}]+(b_{6}+k_{6})[MEKPPERK1P_{n}]-f_{6}[ERK2P_{n}][MEKPP_{n}]+(b_{6}+k_{6})[MEKPPERK2P_{n}]-kd_{8}[MEKPP_{n}][KKPase_{n}]$$

$$\frac{d\left[ MEKPPERK1_{n} \right]}{dt}=NTi_{7}[MEKPPERK1]-NTe_{7}[MEKPPERK1_{n}]+f_{5}[ERK1_{n}][MEKPP_{n}]-(b_{5}+k_{5})[MEKPPERK1_{n}]$$

$$\frac{d\left[ MEKPPERK2_{n} \right]}{dt}=NTi_{7}[MEKPPERK2]-NTe_{7}[MEKPPERK2_{n}]+f_{5}[ERK2_{n}][MEKPP_{n}]-(b_{5}+k_{5})[MEKPPERK2_{n}]$$

$$\frac{d\left[ MEKPPERK1P_{n} \right]}{dt}=NTi_{8}[MEKPPERK1P]-NTe_{8}[MEKPPERK1P_{n}]+f_{6}[ERK1P_{n}][MEKPP_{n}]-(b_{6}+k_{6})[MEKPPERK1P_{n}]$$

$$\frac{d\left[ MEKPPERK2P_{n} \right]}{dt}=NTi_{8}[MEKPPERK2P]-NTe_{8}[MEKPPERK2P_{n}]+f_{6}[ERK2P_{n}][MEKPP_{n}]-(b_{6}+k_{6})[MEKPPERK2P_{n}]$$

$$\frac{d\left[ ELK1 \right]}{dt}=-f_{7}[ERK1PP_{n}][ELK1]+b_{7}[ERK1PPELK1_{n}]-f_{7}[ERK2PP_{n}][ELK1]+b_{7}[ERK2PPELK1_{n}]+kd_{13}[ELK1P][Ptase_{n}]$$

$$\frac{d\left[ ERK1PPELK1_{n} \right]}{dt}=f_{7}\left[ ERK1PP_{n} \right][ELK1]-(b_{7}+k_{7})[ERK1PPELK1_{n}]$$

$$\frac{d\left[ ELK1P \right]}{dt}=k_{7}[ERK1PPELK1_{n}]+k_{7}[ERK2PPELK1_{n}]-f_{8}[ERK1PP_{n}][ELK1P]+b_{8}[ERK1PPELK1P_{n}]-f_{8}[ERK2PP_{n}][ELK1P]+b_{8}[ERK2PPELK1P_{n}]+kd_{12}[ELK1PP][Ptase_{n}]-kd_{13}[ELK1P][Ptase_{n}]+kd_{14}[ELK1PPSRF][Ptase_{n}]$$

$$\frac{d\left[ ERK2PPELK1_{n} \right]}{dt}=f_{7}\left[ ERK2PP_{n} \right][ELK1]-(b_{7}+k_{7})[ERK2PPELK1_{n}]$$

$$\frac{d\left[ ERK1PPELK1P_{n} \right]}{dt}=f_{8}\left[ ERK1PP_{n} \right][ELK1P]-(b_{8}+k_{8})[ERK1PPELK1P_{n}]$$

$$\frac{d\left[ ELK1PP \right]}{dt}=k_{8}[ERK1PPELK1P_{n}]+k_{8}[ERK2PPELK1P_{n}]-f_{9}[ELK1PP][SRF]+b_{9}[ELK1PPSRF]-kd_{12}[ELK1PP][Ptase_{n}]+k_{10}[ELK1PPSRF][mSin3A]$$

$$\frac{d\left[ SRF \right]}{dt}=-f_{9}\left[ ELK1PP \right][SRF]+b_{9}[ELK1PPSRF]+kd_{14}[ELK1PPSRF][Ptase_{n}]+k_{10}[ELK1PPSRF][mSin3A]$$

$$\frac{d\left[ ELK1PPSRF \right]}{dt}=f_{9}\left[ ELK1PP \right][SRF]-b_{9}[ELK1PPSRF]-kd_{14}[ELK1PPSRF][Ptase_{n}]-k_{10}[ELK1PPSRF][mSin3A]$$

$$\frac{d\left[ cFos \right]}{dt}=k_{9}[ELK1PPSRF]$$

## Algebraic equations

The following 5 algebraic equations were used to reduce the number of differential equations:

$$\left[ ERK1 \right]=\left[ ERK1_{tot} \right]-\left[ ERK1P \right]-\left[ ERK1PP \right]-\left[ ERK1n \right]-\left[ ERK1Pn \right]-\left[ ERK1PPn \right]-\left[ MEKPPERK1 \right]-\left[ MEKPPERK1P \right]-\left[ MEKPPERK1n \right]-\left[ MEKPPERK1Pn \right]-\left[ ERK1PPnELK1 \right]-\left[ ERK1PPnELK1P \right]$$

$$\left[ ERK2 \right]=\left[ ERK2_{tot} \right]-\left[ ERK2P \right]-\left[ ERK2PP \right]-\left[ ERK2n \right]-\left[ ERK2Pn \right]-\left[ ERK2PPn \right]-\left[ MEKPPERK2 \right]-\left[ MEKPPERK2P \right]-\left[ MEKPPERK2n \right]-[MEKPPERK2Pn]-\left[ ERK2PPnELK1 \right]-\left[ ERK2PPnELK1P \right]$$

$$[MEK]=\left[ {MEK}_{tot} \right]-\left[ MEKP \right]-\left[ MEKPP \right]-\left[ MEKn \right]-\left[ MEKPn \right]-\left[ MEKPPn \right]-\left[ MEK\boldsymbol{MAPKKK} \right]-\left[ MEKP\boldsymbol{MAPKKK} \right]-\left[ MEKPPERK1 \right]-\left[ MEKPPERK1P \right]-\left[ MEKPPERK1n \right]-\left[ MEKPPERK1Pn \right]-\left[ MEKPPERK2 \right]-\left[ MEKPPERK2P \right]-\left[ MEKPPERK2n \right]-[MEKPPERK2Pn]$$

$$\left[ ELK1 \right]=\left[ ELK_{tot} \right]-\left[ ERK1PPnELK1 \right]-\left[ ERK2PPnELK1 \right]-\left[ ELK1P \right]-\left[ ERK1PPnELK1P \right]-\left[ ERK2PPnELK1P \right]-\left[ ELK1PP \right]-[ELK1PPSRF]$$

$$\left[ SRF \right]=\left[ SRF_{tot} \right]-\left[ ELK1PPSRF \right]$$

# References

| 1. | Huang CYF, and Ferrell JE. Ultrasensitivity in the mitogen-activated protein kinase cascade. Proc. Natl. Acad. Sci. 1996; 93: 10078-10083. |
| --- | --- |
| 2. | Fujioka A, Terai K, Itoh RE, Aoki K, Nakamura T, Kuroda S, et al. Dynamics of the Ras/ERK MAPK cascade as monitored by fluorescent probes. J Biol Chem. 2006;281(13): 8917-8926. |
| 3. | Harrington HA, Komorowski M, Beguerisse-Diaz M, Ratto GM, Stumpf MP. Mathematical modeling reveals the functional implications of the different nuclear shuttling rates of Erk1 and Erk2. Phys Biol. 2012;9(3): 036001. |
| 4. | Radhakrishnan K, Edwards JS, Lidke DS, Jovin TM, Wilson BS, Oliver JM. Sensitivity analysis predicts that the ERK-pMEK interaction regulates ERK nuclear translocation. IET Syst Biol. 2009;3(5): 329-341. |
| 5. | Gille H, Kortenjann M, Thomae O, Moomaw C, Slaughter C, Cobb MH, et al. ERK phosphorylation potentiates Elk-1 mediated ternary complex formation and transactivation. EMBO J. 1995;14(5): 951-962. |
| 6. | Yosimichi G, Nakanishi T, Nishida T, Hattori T, Takano-Yamamoto T, Takigawa M. CTGF/Hcs24 induces chondrocyte differentiation through a p38 mitogen-activated protein kinase (p38MAPK), and proliferation through a p44/42 MAPK/extracellular-signal regulated kinase (ERK). Eur J Biochem. 2001;268(23): 6058-65. |
| 7. | Aplin AE, Stewart SA, Assoian RK, Juliano RL. Integrin-mediated adhesion regulates ERK nuclear translocation and phosphorylation of Elk-1. J Cell Biol. 2001;153(2): 273-82. |
| 8. | Yang SH, Vickers E, Brehm A, Kouzarides T, Sharrocks AD. Temporal recruitment of the mSin3A-histone deacetylase corepressor complex to the ETS domain transcription factor Elk-1. Mol Cell Biol. 2001;21(8): 2802-14. |
| 9. | Kaminska B, Kaczmarek L, Zangenehpour S, Chaudhuri A. Rapid phophorylation of Elk-1 transcription factor and activation of MAP kinase signal transduction pathways in response to visual stimulation. Mol Cell Neurosci. 1999;13(6): 405-14. |
| 10. | Galetic I, Maira SM, Andjelkovic M, Hemmings BA. Negative regulation of ERK and ELK by protein kinase B modulates c-fos transcription. J Bio Chem. 2003;278(7): 4416-23. |
